# Preclinical Efficacy and Safety of an Anti-Human VEGFA and Anti-Human NRP1 Dual-Targeting Bispecific Antibody (IDB0076)

**DOI:** 10.3390/biom10060919

**Published:** 2020-06-17

**Authors:** Jong-Hee Ko, Hyuk-Sang Kwon, Bomin Kim, Gihong Min, Chorong Shin, Seok-Woo Yang, Seong Wook Lee, Youngmin Lee, Dahae Hong, Yong-Sung Kim

**Affiliations:** 1 Research Laboratory, ILDONG Pharmaceutical Co., Ltd., Hwaseong 18449, Korea; kojjong@ildong.com (J.-H.K.); hskwon@ildong.com (H.-S.K.); bm0619@ildong.com (B.K.); mkh3160@ildong.com (G.M.); crshin@ildong.com (C.S.); swyang@ildong.com (S.-W.Y.); seungwook7@ildong.com (S.W.L.); ymlee@ildong.com (Y.L.); dhhong@ildong.com (D.H.); 2 Department of Molecular Science and Technology, Ajou University, Suwon 16499, Korea

**Keywords:** Neuropilin-1, Vascular endothelial growth factor A, IDB0076, angiogenesis, bispecific antibody

## Abstract

Although bevacizumab (Avastin^®^) has been approved as an antiangiogenic agent against some cancers, the efficacy is transient and unsatisfactory in other cancers most likely owing to the presence of alternative proangiogenic factors. Therefore, simultaneous blocking of several proangiogenic factors may be a promising strategy for antiangiogenic cancer therapeutics. Accordingly, neuropilin-1 (NRP1) is an attractive target because it serves as a multifunctional receptor for the vascular endothelial growth factor (VEGF) family. Here, we aimed to generate and test an anti-VEGFA and anti-NRP1 dual-targeting bispecific antibody (named as IDB0076) by genetic fusion of an NRP1-targeting peptide to the C-terminus of the bevacizumab heavy chain. Similar to the parental antibody (bevacizumab), IDB0076 suppressed VEGFA-induced migration of human endothelial cells. In contrast, IDB0076 inhibited endothelial-cell migration induced by other angiogenesis growth factors and manifested a more potent antitumor activity than that of bevacizumab in a murine tumor xenograft model. When toxicity was preliminarily evaluated in cynomolgus monkeys, IDB0076 showed no substantial adverse effects, e.g., the absence of noticeable nephrotoxicity, which has previously been documented for the combination therapy of bevacizumab and an anti-NRP1 antibody. Thus, VEGFA-and-NRP1 dual-targeting bispecific antibody IDB0076 may be a potent and safe anticancer agent worthy of further preclinical and clinical studies.

## 1. Introduction

Angiogenesis, a physiological process of new blood vessel formation on pre-existing vessels, is crucial for tumor growth and metastasis as well as for normal development. Several proangiogenic factors such as the vascular endothelial growth factor (VEGF) family and placental growth factor (PlGF) usually play a major role in the progression of cancer and age-related macular degeneration [1]. The key player of tumor angiogenesis is VEGFA, signaling mainly through VEGF receptor 2 (VEGFR2). A half century of efforts has been dedicated to the development of antiangiogenic agents expected to delay cancer progression by blocking oxygen and nutrient supply to tumor cells [2]. 

Avastin^®^ (bevacizumab) is a recombinant humanized monoclonal antibody that blocks human VEGFA. Due to the proposed universal antitumor activity of bevacizumab, it has been widely tested against advanced and metastatic cancers [3,4,5]. Although such antiangiogenic agents as bevacizumab, Zaltrap^®^ (aflibercept; a recombinant fusion protein), and Cyramza^®^ (ramucirumab; an anti-VEGFR2 monoclonal antibody) have shown some favorable results in terms of increasing overall survival and progression-free survival in several cancers, the benefits to the patients are not satisfactory. Even though an initial response is obtained, resistance develops in the majority of patients [6,7,8,9]. The mechanism of resistance to antiangiogenic agents includes blood vessel formation via the production of alternative proangiogenic factors, e.g., PlGF, angiopoietin, platelet-derived growth factor (PDGF), and hepatocyte growth factor (HGF), and high expression of relevant receptors, VEGFRs, Notch, or neuropilin-1 (NRP1) [10,11]. In numerous clinical trials, combination therapies of antiangiogenic agents have been tested to block these compensatory pathways and to overcome the resistance to antiangiogenic agents [12].

Neuropilin 1) is a transmembrane receptor with several functions related to immunity, development, angiogenesis, and cancer [13]. The receptor interacts with VEGFA and VEGFR2, enhances signaling through this pathway, and promotes angiogenesis [14]. It has been targeted by antibodies [15,16] and peptides [17] to inhibit tumor angiogenesis as well as by peptides as tumor tissue penetration-promoting agents [18]. We recently reported an NRP1-specific peptide, TPP11, which selectively binds to the NRP1-b1b2 domain [19]. Genetic fusion of TPP11 to the C-terminus of the heavy chain of an anti-EGFR antibody (designated as cetuximab-TPP11: Ctx-TPP11) improved tumor homing and tumor tissue penetration of Ctx-TPP11 by loosening cell–cell junctions, as compared to the parental antibody, cetuximab [20,21]. 

Additionally, NRP1 participates in the angiogenesis process by enhancing the binding of PlGF, transforming growth factor beta 1 (TGF-β1), HGF, PDGF, and some fibroblast growth factors (FGFs) to their cognate receptors [13]. As various types of NRP1 blockade can modulate multiple signaling pathways of proangiogenic growth factors, this approach has also been expected to increase antitumor efficacy of some antiangiogenic agents by making tumor vessels vulnerable to anti-VEGF therapy [13,15]. An anti-NRP1 antibody (vesencumab) exerted additive antitumor effects when combined with an anti-VEGF antibody in a preclinical study [15]. Nonetheless, further clinical-trial testing of vesencumab in combination with bevacizumab has failed due to a high incidence of proteinuria [16]. The blockage of both the VEGFA pathway and NRP1 pathway may unnecessarily suppress the VEGF pathway and lead to nephrotoxicity. Even though NRP1 is still a promising target for combination regimens with conventional cancer treatments including bevacizumab, the overlapping sets of adverse effects have become the main obstacle to the development of NRP1-targeted drugs [22,23].

Here, our purpose was to construct and test an anti-VEGFA and anti-NRP1 dual-targeting bispecific antibody (dubbed IDB0076) by genetic fusion of an NRP1-targeting peptide to the C terminus of the heavy chain of the anti-human VEGFA monoclonal antibody (bevacizumab). We demonstrated that IDB0076 simultaneously binds to human VEGFA and human NRP1 in vitro and inhibits the effects of both VEGFA and other angiogenesis growth factors in cell-based assays. Furthermore, repeated treatment with IDB0076 did not induce noticeable systemic adverse effects in cynomolgus monkeys.

## 2. Materials and Methods 

### 2.1. Cells and Reagents

Human umbilical vein endothelial cells (HUVECs; cat. # MC1133) were purchased from Modern Cell & Tissue Technologies (Seoul, Korea). Human BxPC-3 (cat. # CRL-1687), SW620 (cat. # CRL-227), HCT116 (cat. # CRL-247), and SW480 (cat. # CRL-228) cancer cell lines were acquired from the American Type Culture Collection (Manassas, VA, USA). HCT116/Bev-A cells (cat. # 56) and SW480/Bev-A cells (cat. #158), which were developed by Dr. L. M. Ellis [24], were obtained from MD Anderson Cancer Center (Huston, TX, USA). CHO-DG44 cells were kindly provided by Dr. L. Chasin (Columbia University, New York, NY, USA). The EGM-2 Bullet Kit (cat. # CC-3162) and the EBM-2 basal medium (cat. # CC-3156) for cultivation of HUVECs were purchased from Lonza (Basel, Switzerland), whereas RPMI-1640 (for cultivation of BxPC-3 cells, cat. # R7388) from Thermo Fisher Scientific., Inc., (Waltham, MA, USA). McCoy’s 5A medium (for cultivation of HCT116 and HCT116/Bev-A cells; cat. # 16600-082) and Leibovitz’s L-15 medium (for cultivation of SW480 and SW480/Bev-A cells; cat. # 1145-064) were acquired from Sigma-Aldrich (Saint Louis, MO, USA). Cells were confirmed to be free of mycoplasma using the MycoAlert Mycoplasma Detection Kit from Lonza (cat. # LT07-118). Human VEGFA 165 (cat. # 293-VE), the human NRP1 extracellular domain (ECD; cat. #3870-N1-025), the mouse NRP1 ECD (cat. # 5994-N1-050), PlGF2 (cat. # 6837-PL-025), and VEGFB (cat. # 751-VE) were purchased from R&D Systems (Minneapolis, MN, USA), and fluorescein isothiocyanate (FITC)-labeled dextran (40 kDa, cat. # FD40S) from Sigma-Aldrich.

### 2.2. Protein Expression and Purification

The heavy chain and light chain of IDB0076 were cloned into the pPGXII vector (PanGen Biotech, Suwon, Korea) at NheI/XhoI restriction sites, separately. These plasmids were transfected into CHO-DG44 cells using microporator-mini (Thermo Fisher). The expressed protein was purified by means of Hiscreen Mabselect^TM^ SuRe^TM^ (GE Healthcare, Chicago, IL, USA) and analyzed by sodium lauryl sulfate (SDS-PAGE) and size exclusion chromatography (SEC). The SDS-PAGE analysis was performed according to the instructions of the manufacturer (Bio-Rad, Hercules, CA, USA) in a 4–20% precast Tris-glycine gel. The proteins were analyzed by SEC on a Biosuite 250 (4 μm, 4.6 × 300 mm) UHR SEC column (Waters, Milford, MA, USA) equilibrated with a buffer consisting of 200 mM potassium phosphate and 250 mM KCl (pH 6.2) at a flow rate of 0.35 mL/min.

### 2.3. Affinity Measurement by Surface Plasmon Resonance Spectroscopy

Affinity levels of IDB0076 for VEGFA and for the NRP1 ECD were measured using a Biacore T200 instrument (GE Healthcare). The ligand VEGFA was immobilized on a CM5 chip at the level of 16 response unit (RU), and IDB0076 or bevacizumab was diluted and injected into the VEGFA-coated chip at a concentration of 6.25–200 nM for the analysis. In the NRP1-binding assays, the human NRP1 ECD or mouse NRP1 ECD was immobilized on CM5 chips at the level of 1600 or 1800 RU, respectively. IDB0076 was diluted and injected into the NRP1 ECD-coated chip at a concentration of 0.156–20.000 nM. Kinetic parameters were determined in the Biacore T200 evaluation software, ver. 3.0 (GE Healthcare). The Langmuir 1:1 model was employed for the binding analysis.

### 2.4. A Wound Healing Assay

Human umbilical vein endothelial cells were seeded in six-well plates (3 × 10^4^ cells/well) and incubated in EGM-2 with 10% fetal bovine serum for two days, followed by serum starvation for 6 h. After that, a straight scratch was made across the layer of cultured cells with a 200 μL micropipette tip. The cells were treated with bevacizumab, aflibercept, or IDB0076 in the presence or absence of VEGFA (20 ng/mL), VEGFB (50 ng/mL), or PlGF2 (50 ng/mL) in EBM-2 (0.5% FBS) and were incubated at 37 °C and 5% CO_2_. Images of the cells were obtained with Eclipse TE2000-U (Nikon, Tokyo, Japan) at 0 h and at the final hour (14–16 h). The wound area was quantitated in the Image J software, ver. 1.4.3.67 (National Institutes of Health, Bethesda, MA, USA).

### 2.5. A Permeability Assay

Permeability across an endothelial monolayer was examined using HUVECs. These cells were seeded (2 × 10^5^ cells/well) into a 24-well Transwell chamber (0.4 μm pore size, 6.5 mm, polycarbonate; Corning Costar) and grown for three days until the formation of a tight monolayer. The cells were then serum starved for 4 h, and VEGFA (50 ng/mL), bevacizumab (1 μM), or IDB0076 (1 μM) was added to the upper chamber and lower chamber and incubated overnight. After that, 10 μg of fluorescein isothiocyanate (FITC)-labeled dextran was added into the upper chamber. After 1 h treatment, the medium of the lower compartment was analyzed on a Synergy H4 Hybrid fluorescent plate reader (BioTek, Winnooski, VT, USA). Data were normalized to PBS-treated samples.

### 2.6. A Tube Formation Assay

To prepare conditioned media (CMs) from cancer cells (HCT116, HCT116/Bev-A, SW480, and SW480/Bev-A cells), these cells were cultured with each basal medium plus 1% of FBS for 24 h, followed by treatment with vehicle, bevacizumab, or IDB0076. PBS was used as vehicle. After 24 h, the CM was collected and centrifuged to remove cell debris. The human endothelial cells were seeded on μ-slide angiogenesis tool (ibidi, Fitchburg, WI, USA)-coated Matrigel (Corning Life Sciences, Corning, NY, USA) with 50 μL of one of these CMs. At 24 h after the seeding, images were captured under a light microscope. Total tube length was measured and analyzed with an angiogenesis analyzer (Image J software).

### 2.7. A Pharmacokinetic (PK) Assay

This animal experiment was approved by the Animal and Ethics Review Committee of ILDONG Pharmaceutical, Co., Ltd. (Hwaseong, Korea) and was performed according to the guidelines established by the Institutional Animal Care and Use Committee (IACUC; approval No. 1808-4). Sprague–Dawley (SD) rats (males, eight weeks old) were acquired from Orient Bio Inc. (Seongnam, Korea). Five SD rats were given a 10 mg/kg intravenous (i.v.) dose of either IDB0076 or bevacizumab, and blood samples (approximately 0.25 mL each) were collected at 5 min and 1, 2, 4, 8, 24, 32, 72, 120, 192, 240, and 360 h post-injection. The samples were centrifuged at 4 °C and 13,475 g for 5 min, and serum was collected and stored at −80 °C until analysis. The concentration of IDB0076 or bevacizumab in each serum sample was determined by two types of enzyme-linked immunosorbent assay (ELISA). One is a VEGFA-binding ELISA, where a target drug binds to VEGFA and is detected by means of an anti-human IgG–horseradish peroxidase (HRP) conjugate (cat. # A7164, Sigma Aldrich) to evaluate the binding affinity for VEGFA. The other is a dual-targeting ELISA, where a target drug binds to VEGFA and is detected by means of a biotinylated NRP1 b1b2 domain to test whether IDB0076 is capable of binding to both targets simultaneously. The NRP1 b1b2 domain was produced and purified as described previously [17], and the biotinylation was conducted according to the manufacturer’s instructions (EZ-Link micro sulfo-NHS-biotinylation Kit, cat. # 21925, Thermo Fisher Scientific Inc.). The PK parameters (AUC_all_, AUC_inf_, t_1/2_, C_0_, CL, and V_ss_) were estimated by noncompartmental analysis in the Pheonix^TM^ WinNonlin^®^ software, ver. 7.0 (Pharsight, Princeton, NJ, USA) and were presented as mean ± standard deviation.

### 2.8. In Vivo Antitumor Activity of IDB0076

This animal experiment was evaluated and approved by the Animal and Ethics Review Committee of Ajou University (Suwon, Korea) and was performed according to the guidelines established by the IACUC (approval No. 2017-0011). BALB/c nude mice (females, four weeks old) were acquired from NARA Biotech (Seoul, Korea). The BxPC-3 cells (5 × 10^6^ cells/mouse) resuspended in 50% Matrigel (BD Biosciences, San Jose, CA, USA) were inoculated subcutaneously into the right flank of the mice. When the mean tumor volume reached 150 mm^3^, the mice were randomized into five groups (vehicle, 10 mg/kg bevacizumab, 1 mg/kg IDB0076, 3 mg/kg IDB0076, and 10 mg/kg IDB0076) and were i.v. injected with these agents via the tail vein twice a week for four weeks. Tumors were measured with calipers, and tumor volumes were estimated via the formula: length × (width)^2^/2. Body weight of the mice was measured twice a week. After all the mice were euthanized, each tumor was excised, fixed in formalin, and embedded in paraffin. The paraffin blocks were cut at a thickness of 4 μm. Endothelial cells were labeled with a rat monoclonal anti-CD34 antibody (cat. # ab8158, Abcam, Cambridge, UK). Pericytes were labeled with a rabbit polyclonal anti-NG2 antibody (cat. # ab5320, Merck, Darmstadt, Germany). Some samples were double-stained with an anti-CD34 antibody and anti-NG2 antibody (*n* = 7 per group). The secondary antibody was either a goat anti-rabbit IgG Alexa Fluor 488-conjugated antibody (cat. # A27034, Thermo Fisher Scientific Inc.) or a goat anti-rat IgG Alexa Fluor 594-conjugated antibody (cat. # A11007, Thermo Fisher Scientific Inc.). Images were captured via confocal microscopy (Carl Zeiss, Thornwood, NY, USA) and were subjected to Zen 2.3 Blue edition analysis (Carl Zeiss).

### 2.9. Toxicity Evaluation

A 4-week toxicity assessment in cynomolgus monkeys was conducted at Shin Nippon Biomedical Laboratories, Ltd. (SNBL, Tokyo, Japan). The protocol of this experiment was approved by the IACUC (approval No. IACIC436-001) and was performed in accordance with the animal welfare bylaws of SNBL, Drug Safety Research Laboratories, which is accredited by AAALAC International. The purpose of the experiment was to investigate the toxicity of IDB0076 when administered to cynomolgus monkeys by i.v. injection twice a week for four weeks, followed by a 4-week recovery period. This experiment involved four monkeys per sex, aged between three and four years and weighing between 2.68 and 3.12 kg. The monkeys were randomly subdivided as follows: Group 1 (low dose, 2 mg/kg) and Group 2 (medium dose, 10 mg/kg) contained one animal per sex per group, and Group 3 (high dose, 50 mg/kg) contained two animals per sex per group. At the end of the dosing period, Group 1, Group 2, and one animal/sex from Group 3 were necropsied. The remaining two animals in Group 3 remained untreated for four weeks. At the end of the recovery period, they were necropsied. All the animals were examined daily regarding deaths and their general condition. The body weight of each monkey was determined on Day −1, weekly during the experiment, and on the day of necropsy. Food consumption was assessed daily during the experiment. Urinalysis was conducted three times in total: before the test and prior to the terminal and recovery necropsies by means of an automated urine chemistry analyzer (Clinitek Atlas XL, Sparton Medical Systems, Schaumburg, IL, USA) and an automatic analyzer (JCA-BM6070, JEOL Ltd., Tokyo, Japan). The organs were weighed, and then relative organ weights per kilogram of body weight were calculated from the body weight on the day of necropsy. Hematological and biochemical parameters were evaluated on a hematology analyzer (XT-2000iV, Sysmex corporation, Kobe, Japan) and the automatic analyzer (JCA-BM6070), respectively. For histopathological examination, testes were fixed in a formalin–sucrose–acetic acid solution, while other organs and tissues were fixed in 10% neutral buffered formalin. The femur and femoral bone marrow were decalcified with Kalkitox (FUJIFILM Wako Pure Chemical Corporation, Osaka, Japan). Electron-microscopic examination of kidney glomeruli was carried out under a transmission electron microscope (JEM-1400Plus, JEOL Ltd.) at the end of dosing and at the end of the recovery period. 

### 2.10. Statistical Analysis

Data are reported as means ± standard error of the mean (SEM) unless specified otherwise. A comparison of data from test groups and controls was made to assess statistical significance by two-tailed, unpaired Student’s *t*-test in Microsoft Excel 2016.

## 3. Results

### 3.1. Preparation and Characterization of IDB0076

**Figure 1:**
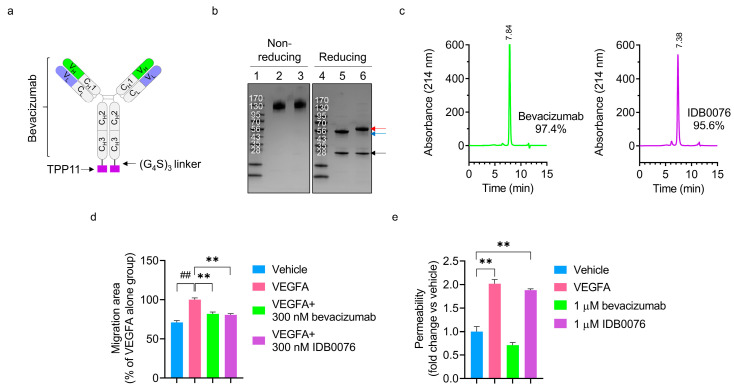
IDB0076 was produced in CHO-DG44 cells and characterized in comparison with bevacizumab. (**a**) The schematic diagram of IDB0076, in which TPP11 was fused via a 15-residue (G_4_S)_3_ linker to the C-terminus of bevacizumab. (**b**) IDB0076 was compared to bevacizumab by SDS-PAGE analysis under nonreducing conditions and reducing conditions. Red and blue arrows indicate the heavy chain of IDB0076 and bevacizumab, respectively. The black arrow points to the light chain of IDB0076 and bevacizumab. Lanes 1 and 4: markers, lanes 2 and 5: bevacizumab, and lanes 3 and 6: IDB0076. (**c**) IDB0076 and bevacizumab were characterized by size exclusion high-performance liquid chromatography with monitoring at 214 nm. The left column: bevacizumab, the right column: IDB0076. (**d**) The vascular endothelial growth factor A (VEGFA)-induced migration assay was performed on human umbilical vein endothelial cells (HUVECs) to determine the biological function of IDB0076 in comparison with bevacizumab. Under starvation, HUVEC monolayers were scratched and treated with either 300 nM IDB0076 or 300 nM bevacizumab in the presence of VEGFA. Then, the area of migration into the scratch was determined. Data are presented as mean ± SEM (*n* = 3); ^##^ *p* < 0.01 as compared with the vehicle group, ** *p* < 0.01 as compared with the VEGFA-alone group. (**e**) IDB0076 increases permeability of an endothelial-cell monolayer as compared with bevacizumab. Permeability across the HUVEC monolayer was assessed by fluorescein isothiocyanate (FITC)-dextran passage after the cells were stimulated with VEGFA (50 ng/mL), bevacizumab (1 μM), or IDB0076 (1 μM) overnight. After the incubation, FITC-dextran was applied to the upper chamber for 1 h incubation. FITC-dextran fluorescence values were compared with those of vehicle. Data are presented as mean ± SEM (*n* = 3); ** *p* < 0.01 as compared with the vehicle group.

The IDB0076 was generated by genetic fusion of NRP1-targeting peptide TPP11 (HTPGNSKPTRTPRR) to the C-terminus of the heavy chain of bevacizumab via the (G_4_S)_3_ linker (Figure 1a). The IDB0076 was successfully expressed by transfection into CHO-DG44 cells and was purified on an affinity column. The heavy chain of IDB0076 was larger than that of bevacizumab according to SDS-PAGE under reducing conditions, owing to the addition of TPP11 via the linker (Figure 1b). IDB0076 was found to assemble into a homodimer, just as bevacizumab does, under nonreducing conditions. To confirm purity in comparison to bevacizumab, SEC was performed under nondenaturing conditions (Figure 1c). Purified IDB0076 and bevacizumab contained some high-molecular-weight aggregates even though their purity was above 95%. These high-molecular-weight aggregates may stem from bevacizumab because these results are consistent with the findings of prior bevacizumab research [25]. These data confirmed that IDB0076 was prepared in the correctly assembled form with high purity.

### 3.2. Assays of IDB0076 Binding

The binding affinity of IDB0076 for VEGFA was analyzed using a surface plasmon resonance-based biosensor (Table 1). Equilibrium dissociation rate constant, *K*_D_, values of IDB0076 and bevacizumab toward VEGFA turned out to be 1.202 × 10^−9^ M and 1.438 × 10^−9^ M, respectively. The binding affinity for VEGFA was almost the same between IDB0076 and bevacizumab, which means that the TPP11 fusion should not alter the affinity of the anti-human VEGF antibody for VEGFA. We then evaluated the biological function of IDB0076 in the VEGFA-induced HUVEC migration assay (Figure 1d). After 14 h of treatment, migration of HUVECs was significantly inhibited by IDB0076 as compared to vehicle, during administration of 20 ng/mL VEGFA. This result was very similar to that on bevacizumab and suggested that IDB0076 has full bioactivity in terms of VEGFA blockage. We also measured the binding of IDB0076 to the human and mouse NRP1 ECD. The binding affinity of IDB0076 for the human and mouse NRP1 ECDs was found to be 1.574 × 10^−8^ M and 2.197 × 10^−8^ M, respectively. These values are 10-fold weaker than the above-mentioned affinity for VEGFA (Table 1). These results meant that VEGFA-and-NRP1 dual-targeting IDB0076 binds 10-fold more strongly to VEGFA than to NRP1.

We have previously reported that immunoglobulin Fc-fused TPP11 (Fc-TPP11) enhances vascular permeability and increases paracellular permeability in tumor tissues by inducing cellular internalization of NRP1 [19]. To test whether IDB0076 still had the biological activity, we conducted the HUVEC permeability assay and the NRP1 internalization assay in cancer cells. IDB0076 enhanced the passage of FITC-dextran through HUVEC monolayers by 1.9-fold, similarly to the effect of VEGFA (Figure 1e). Stimulation of IDB0076 to cell-surface expressed NRP1 efficiently induced cellular internalization in cancer cells in comparison with parent antibody, bevacizumab (Supplementary Figure S1). These results indicated that IDB0076 triggers cellular internalization of NRP1 through the TPP11 moiety.

### 3.3. Inhibition of Multiple Signaling Pathways of Proangiogenic Growth Factors by IDB0076 

**Figure 2:**
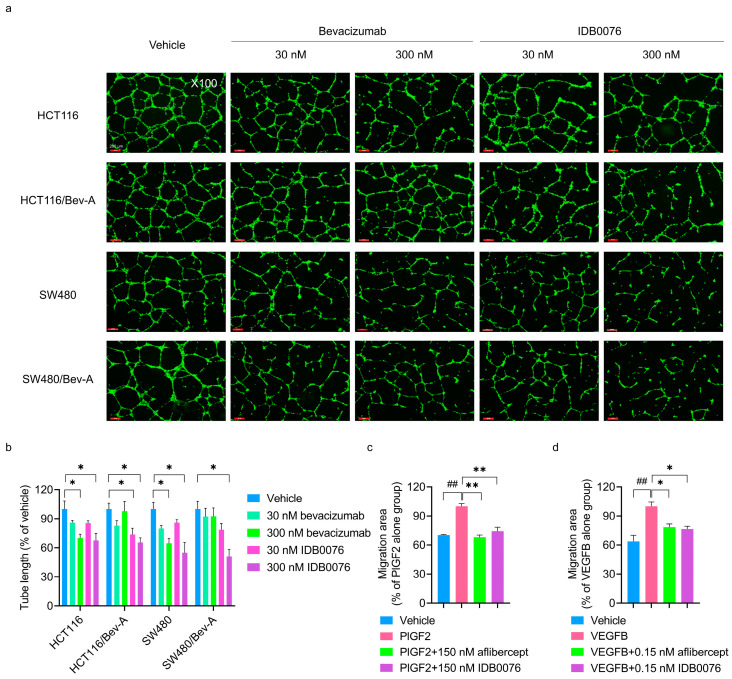
IDB0076 suppresses angiogenic effects of multiple proangiogenic growth factors in human umbilical vein endothelial cells (HUVECs). (**a**,**b**) IDB0076 inhibited the tube formation by HUVECs stimulated by the conditioned medium (CM) derived from the parental cells (HCT116 and SW480 cells) or bevacizumab-adapted cells (HCT116/Bev-A and SW480/Bev-A cells) treated with vehicle, IDB0076, or bevacizumab for 24 h, followed by the induction of tube formation for 24 h. Representative images out of three independent experiments are shown. The results were quantified by measurement of tube length using an angiogenesis analyzer. Data are presented as mean ± SEM (*n* = 3); * p < 0.05 as compared with the vehicle group treated with the CM derived from each cancer cell line. Scale bars = 200 μm, ×100 magnification. (**c**,**d**) IDB0076 inhibits PlGF2- or VEGFB-induced HUVEC migration comparably to aflibercept. Under starvation, cell monolayers were scratched and treated with the indicated concentration of IDB0076 or aflibercept in the presence of either PlGF2 or VEGFB. After that, the area of migration into the scratch was determined. Data are presented as mean ± SEM (*n* = 3); *^##^ p* < 0.01 as compared with the vehicle group; * p < 0.05, ** p < 0.01 as compared with the growth factor alone group.

Neuropilin-1 acts as a coreceptor for a number of extracellular ligands such as semaphorins, VEGFs, and other proangiogenic growth factors [14]. Ellis et al. have reported that chronic exposure to bevacizumab facilitates tumor cell migration by means of compensatory pathways in colorectal cancer cells [24]. These bevacizumab-adapted cells secrete more VEGFA, VEGFB, VEGFC, and PlGF, which induce migration of tumor cells and angiogenesis of endothelial cells in this medium in comparison with the parental cells [24]. We determined whether IDB0076 could suppress the function of proangiogenic growth factors in HUVECs by competitive binding to NRP1. Human endothelial cell tube formation was induced by a CM from bevacizumab-adapted cells (HCT116/Bev-A and SW480/Bev-A cells), which secrete a variety of proangiogenic growth factors [24], or by a CM from parental cells (HCT116 and SW480 cells). IDB0076 and bevacizumab inhibited HUVEC tube formation induced by the parental-cell CM relative to vehicle at a concentration of 300 nM. IDB0076 also caused concentration-dependent inhibition of tube formation by HUVECs stimulated by the bevacizumab-adapted cell CM. In contrast, bevacizumab treatment failed to inhibit HUVEC mobility induced by the bevacizumab-adapted cell CM (Figure 2a,b). These results meant that IDB0076 can suppress signaling of other proangiogenic growth factors besides VEGFA and can break the resistance to bevacizumab by blocking the activity of these growth factors.

Furthermore, we examined the effects of IDB0076 on VEGFB- or PlGF2-induced migration of HUVECs as compared to aflibercept. The latter is a recombinant Fc fusion protein composed of the binding domains of VEGFR1 and VEGFR2 and traps VEGF family members such as VEGFA, VEGFB, and PlGF. The addition of either PlGF2 or VEGFB significantly increased HUVEC migration as compared to a no-growth factor control group. IDB0076 significantly attenuated this migration under the influence of PlGF2 or VEGFB to the level similar to that of the group treated with aflibercept (Figure 2c,d). These data suggested that IDB0076 might be a potent blocker of VEGF family ligands via binding to NRP1, although IDB0076 cannot trap the proangiogenic growth factors directly.

### 3.4. Pharmacokinetics of IDB0076

**Figure 3:**
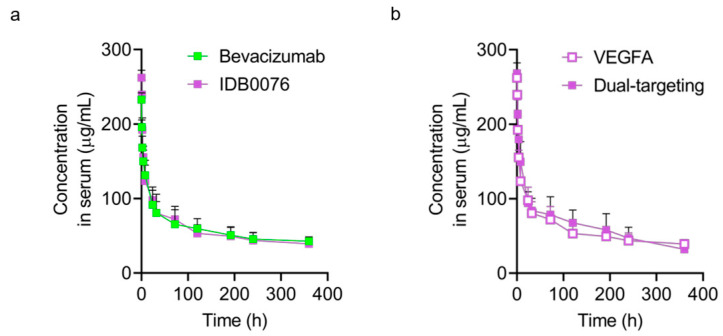
IDB0076 retains the binding affinity for VEGFA and NRP1 simultaneously in the pharmacokinetic (PK) assay. (**a**) Sprague–Dawley (SD) rats received a single i.v. injection of 10 mg/kg IDB0076 or bevacizumab via the tail vein. The serum concentrations of IDB0076 and bevacizumab are presented, as determined based on their binding activity for immobilized human VEGFA in an enzyme-linked immunosorbent assay. (**b**) The serum concentration of IDB0076 was measured based on its binding activity for immobilized VEGFA and was determined by means of the biotinylated NRP1 b1b2 domain. Each symbol and error bar represent the arithmetic mean and the standard deviation of each drug at a given time point (*n* = 5).

The PK assay of IDB0076 was performed on SD rats in comparison with bevacizumab. Semi-log plots of serum concentration of IDB0076 or bevacizumab versus time after a single i.v. dose (10 mg/kg) are presented in Figure 3. When determined by the VEGFA-binding ELISA, serum concentration–time profiles of IDB0076 and bevacizumab were similar (Figure 3a). Furthermore, none of the PK parameters had significant differences between the IDB0076 group and bevacizumab group (Table 2). Half-life (t_1/2_) of IDB0076 and bevacizumab was 14.7 ± 2.0 and 14.7 ± 1.8 days, respectively, i.e., within the range of values reported in another study on bevacizumab (12.3 ± 3.2 days) [26]. These results showed that the PK parameters and VEGFA-binding affinity of IDB0076 are comparable to those of the parental antibody. We next determined whether IDB0076 can bind to VEGFA and NRP1 simultaneously in the sandwich ELISA (dual-targeting ELISA). Similar time profiles of IDB0076 in the VEGFA-binding ELISA and dual-targeting ELISA indicated that during circulation in the blood stream, TPP11 did not split from the heavy chain of the antibody or lose the binding affinity for NRP1 and supported the half-life of the antibody (Figure 3b).

### 3.5. Antitumor Activity of IDB0076 in a Pancreatic Cancer Xenograft Model

**Figure 4:**
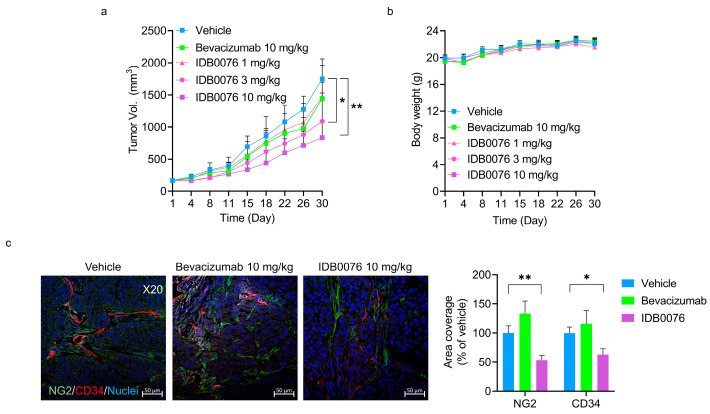
IDB0076 suppressed tumor growth and neovascularization in the BxPC-3 xenograft model in comparison with bevacizumab. Mice were inoculated subcutaneously into the right flank with 5 × 10^6^ BxPC-3 cells. The mice bearing BxPC-3 cells were dosed with vehicle, IDB0076, or bevacizumab i.v. twice a week for 4 weeks. (**a**,**b**) Tumor growth and body weight were measured and monitored twice a week. Data are presented as mean ± SEM (*n* = 7 per group). (**c**) Representative images of blood vessels stained for CD34 (red), NG2 (green), and nuclei (blue). The bars in the graph denote the quantification of blood vessel and pericyte coverage in comparison with vehicle as the mean ± SEM of 10 images per tumor (*n* = 7 per group). Magnification: ×20, scale bar: 50 μm; * *p* < 0.05, ** *p* < 0.01 as compared with the vehicle group.

There are few effective biologics for patients with pancreatic ductal adenocarcinoma (PDAC), which is the fourth leading cause of cancer-related deaths in the world [27]. Although PDAC is known to secrete a variety of proangiogenic growth factors, most patients with PDAC do not manifest any response to antiangiogenic agents [28,29]. We have already demonstrated that TPP11 renders cancer cells susceptible to anti-EGFR therapy in PDAC xenograft models [20]. In the present study, we determined whether IDB0076 has additive effects relative to anti-VEGF therapy in the PDAC xenograft model. IDB0076 was evaluated for its antitumor ability in the BxPC-3 xenograft model, as compared with bevacizumab. Although bevacizumab treatment only negligibly inhibited the tumor growth in BxPC-3-bearing mice, IDB0076 treatment significantly retarded this tumor growth in a dose-dependent manner (Figure 4a). The analysis on day 30 revealed that the effects of bevacizumab treatment at a dose of 10 mg/kg biweekly were not statistically significantly different from those in the vehicle group. By contrast, IDB0076 treatment yielded 52% tumor growth inhibition at the same dose. No significant difference in body weight was noted between the mice treated with IDB0076 and those treated with bevacizumab in this model (Figure 4b). To further evaluate the intratumoral vascular changes caused by the treatment with IDB0076 or bevacizumab in the BxPC-3 xenograft model, tumors were collected at the same time for histological analysis. In the bevacizumab-treated group, vascular coverage did not diminish, whereas both blood vessel coverage and pericyte coverage were significantly lower, by 46.4% and 37.1%, respectively, in the 10 mg/kg IDB0076-treated group than in the vehicle group (Figure 4c). 

### 3.6. Toxicity of IDB0076 in Cynomolgus Monkeys

**Figure 5:**
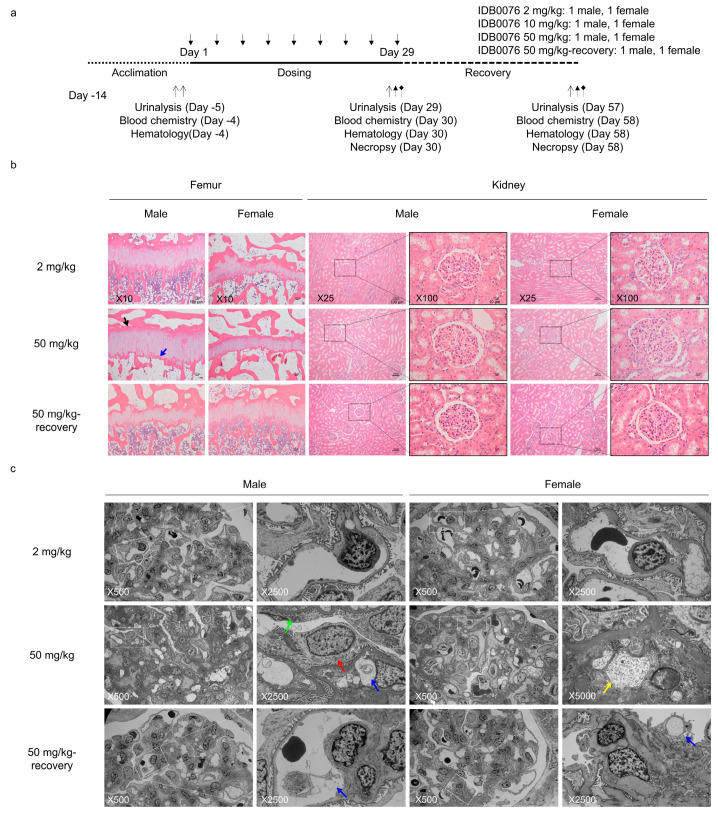
Toxicity of IDB0076 was investigated in cynomolgus monkeys at doses of 2, 10, and 50 mg/kg during 4 weeks. (**a**) Experimental design for the evaluation of IDB0076 toxicity in cynomolgus monkeys. (**b**) Light-microscopic appearance of the femur and kidneys from monkeys treated with IDB0076 was examined at the end of dosing and after the end of the recovery period. Black and blue arrows indicate the thickness of the physis and the primary trabecular bone, respectively (magnification: ×10). The kidney section illustrates a glomerulus at lower (×25) and higher magnification (×100). (**c**) Electron-microscopic images of glomeruli from monkeys treated with IDB0076 at the end of dosing and after the end of the recovery period. Representative images of a glomerulus are shown at lower (×500) and higher magnification (×2500). Granular/vesicular material in mesangial cells was detected in females at the 50 mg/kg dose (×5000 magnification). Each colored arrow indicates a change of the glomerulus as follows: red, hypertrophy of podocytes; green, shortening of foot processes in the podocytes; blue, retention of vesicles in the capillary; yellow, granular/vesicular material in mesangial cells.

To investigate IDB0076 toxicity, a 4-week toxicity assessment in cynomolgus monkeys was conducted. IDB0076 was administered i.v. twice weekly for 4 weeks at a dose of 2, 10, or 50 mg/kg to one male and one female monkey per group (Figure 5a). One male and one female were added to the highest dose group to assess the reversibility and persistence of any effects after a 4-week recovery period. During the dosing period, no animal died or was euthanized due to morbidity, and no IDB0076-related changes were noted in clinical signs, body weight, food consumption, hematological parameters, organ weight, or necropsy findings in any group (Supplementary Tables S1 and S2). At 50 mg/kg, increased globulin levels and a decreased albumin/globulin ratio were noted when compared with the predose value. Increased total-cholesterol concentration in the blood was observed only in females at 50 mg/kg (Table 3). Nevertheless, these changes were found to be reversed by the end of the recovery period. They were assumed to be toxicologically insignificant because no corresponding histopathological lesions were observed (Table 4). 

Increased thickness of the physis and a decreased amount of primary trabecular bone were detected in the femur at 10 and 50 mg/kg doses of IDB0076 (Table 4, Figure 5b). Enlargement of glomeruli was observed in kidneys at 50 mg/kg (Table 4, Figure 5b). It correlated with an elevated level of total protein in urine in the monkeys treated with the same dose of IDB0076 (Table 5). To investigate the impact of IDB0076 on enlargement of the glomeruli, electron-microscopic examination was performed on the males and females at 50 mg/kg as compared with the 2 mg/kg-treated group, which was found to be normal by light microscopy. The glomerular changes in kidneys at 50 mg/kg consisted of podocyte hypertrophy, shortening of foot processes in the podocytes, retention of small and large vesicles in capillaries, and granular/vesicular material in mesangial cells (Figure 5c, Table 4). By contrast, either these glomerular and femur changes disappeared or their incidence and severity decreased after the 4-week recovery period. Thus, the no-observed-adverse-effect level was assumed to be 2 mg/kg in males and 10 mg/kg in females. The highest non-severely toxic dose was estimated to be 50 mg/kg for both sexes. 

## 4. Discussion

Combination therapy with antiangiogenic agents is a suitable strategy to overcome the limitations of existing antiangiogenic agents through blocking of compensatory signaling pathways [12]. The biggest hurdle for the development of combination therapies of different antiangiogenic agents is the narrow safety margin because of overlapping sets of adverse effects such as hypotension, proteinuria, impaired wound healing, hemorrhage, and thrombosis even though the effectiveness of the combination is better than that of a single agent [30]. Various types of NRP1 blockade are considered a promising combination partner for conventional antiangiogenic agents because an anti-NRP1 antibody has additive effects with an anti-VEGF agent against cancer by enhancing the antiangiogenic action [15]. Nonetheless, the combination of the anti-NRP1 antibody (vesencumab) with bevacizumab failed the testing in clinical studies because of severe nephrotoxicity, which is ascribed to strong inhibition of VEGF pathways [16,31]. We have hypothesized that IDB0076 is better tolerated than the combination of an anti-NRP1 antibody with bevacizumab owing to the lower affinity of IDB0076 for NRP1 as compared to the anti-NRP1 antibody [32]. To ascertain additional adverse effects of IDB0076, we evaluated its toxicity in detail in cynomolgus monkeys. Hypertrophy of podocytes and shortening of foot process in glomeruli were noted at a high dose that caused an increase in urine protein levels by changing the gap in the filtration slit (Figure 5, Table 4 and Table 5). This nephrotoxicity has been detected in VEGFA knockout mice or mice treated with an anti-mouse VEGFA antibody [23,31]. These changes in our study were assumed to be IDB0076-related toxicological effects because of the blockage of VEGFA signaling, but these changes were detected only at 50 mg/kg and seemed to be reversible. Other toxicological changes here, including femur changes under the influence of IDB0076, were largely consistent with findings about bevacizumab in a repeat toxicity study in cynomolgus monkeys [33,34]. Prior to the present toxicity evaluation, we tested whether the NRP1-binding property of IDB0076 can persist fully in vivo during PK and anticancer efficacy assays. IDB0076 retained the binding affinity for NRP1 and for VEGFA during circulation in the blood for 15 days (Table 2), suggesting that the NRP1-binding TPP11 moiety fused to the C terminus of the heavy chain of IDB0076 is not cleaved by peptidases in serum. In the BxPC-3 xenograft model here, the VEGFA-and-NRP1 dual-targeting bispecific antibody (IDB0076) significantly retarded the tumor growth and tumor blood vessel formation as compared with the VEGFA-blocking monospecific antibody bevacizumab (Figure 3). The above results indicate that the cynomolgus monkeys in the toxicity experiment were sufficiently exposed to IDB0076 having a full biological activity. In this study, we demonstrated that IDB0076 blocks VEGFA and NRP1 simultaneously and does not show much cause for concern about overlapping sets of adverse effects in future clinical studies. Numerous preclinical studies should be conducted in nonhuman primates, who are pharmacologically relevant species in relation to bevacizumab and the anti-NRP1 antibody for optimizing the strength of suppression of VEGF-related pathways by the combination of bevacizumab and the anti-NRP1 antibody. In contrast, the development of IDB0076 has advantages for preclinical and clinical testing in terms of predicted safety in humans as compared to the combination therapy because IDB0076 is a bispecific antibody blocking VEGFA and NRP1 as monotherapy.

It has already been reported that NRP1 regulates internalization and trafficking through endosomal transport pathways of a number of cell surface receptors and their receptor–ligand complexes [35]. In our previous study, we have demonstrated that cellular internalization of NRP1 in response to TPP11 is essential for inducing permeability [17]. In addition, we have also reported that Ctx-TPP11 internalization by cancer cells is dependent on NRP1 and that Ctx-TPP11 suppresses active integrin β1-driven signaling through NRP1-coupled internalization of integrin β1 [20]. Here, we report that IDB0076 suppresses various pathways of proangiogenic growth factors in a HUVEC tube formation assay involving a CM from bevacizumab-adapted cancer cells, which secrete a variety of proangiogenic growth factors (Figure 2). We also found that IDB0076 inhibits VEGFB- and PlGF2-induced endothelial-cell migration comparably to the action of aflibercept, which directly traps VEGF family members. Our data indicate that IDB0076 may also trigger cointernalization of a cognate receptor (VEGFR1 for VEGFB and PlGF2) by inducing cellular internalization of NRP1 and should break the resistance to bevacizumab by blocking these growth factors taking part in tumor angiogenesis. Yaqoob et al. reported that NRP1 stimulates tumor growth by increasing α5β1 integrin-dependent fibronectin fibril assembly in the tumor microenvironment [36]. Besides α5β1 integrin, NRP1 co-interacts EGF with EGFR, and HGF with c-Met, and activates these pathways which participate in the proliferation of cancer cells [13]. Li et al. had demonstrated that depletion of NRP1 counteracted EGF-induced EGFR activation and attenuated HGF-stimulated c-Met signaling pathway in gastric cancer cells [37]. As IDB0076 decreases NRP1 expression in cell surface via NRP1 internalization, IDB0076 may inhibit the above pathways and retard more tumor growth than blocking VEGFA alone. Further studies are under way to directly investigate the mechanism which IDB0076 suppresses multi-signal pathways dependent on the expression of NRP1 and co-receptors in various cancer cells via NRP1-coupled internalization.

Here, we demonstrated that IDB0076 offers some advantages in terms of penetration into a tumor and blocking of multiple pathways of proangiogenic factors with manageable toxicity. Owing to these favorable properties, IDB0076 may be beneficial to patients with malignant PDACs, colorectal cancers, and breast cancers, which block deep and wide penetration of anticancer drugs into tumor tissues because of an extensive desmoplasia microenvironment or because these cancers are not susceptible to bevacizumab monotherapy owing to compensatory pathways. Besides, recent studies proved that VEGF inhibitors convert a tumor microenvironment from an immunosuppressive to immunosupportive one and help to overcome the low response rate of cancers to the treatment with immune checkpoint inhibitors [38]. It is also well documented that NRP1 plays important roles in the function and stability of intratumoral regulatory T cells [13]. Furthermore, we demonstrated that TPP11 selectively inhibits regulatory T cell function only in a tumor microenvironment in a very recent study [39].

## 5. Conclusions

These results suggest that IDB0076 may have synergistic effects when combined with immune checkpoint inhibitors as well as chemotherapy by blocking both VEGFA and NRP1. Consequently, if we see a favorable safety profile of IDB0076 in many toxicological studies, then IDB0076 can enter clinical development as a safe and new promising anticancer agent. Additionally, follow-up development of IDB0076 will further increase the value of NRP1 as an anticancer target.

## Figures and Tables

**Table 1:** Binding affinity of IDB0076 for VEGFA and neuropilin-1 (NRP1) according to the Biacore system.

Immobilized Antigen		Analyte	*k_a_* (M^−1^s^−1^)	*k_d_* (s^−1^)	*K*_D_ (M)
Protein	Species				
NRP1 ECD	Human	IDB0076	1.221 × 10^5^	1.921 × 10^−^^3^	1. 574 × 10^−^^8^
NRP1 ECD	Mouse	IDB0076	1.885 × 10^5^	4.142 × 10^−^^3^	2.197 × 10^−^^8^
VEGFA	Human	IDB0076	2.904 × 10^4^	3.491 × 10^−^^5^	1.202 × 10^−^^9^
VEGFA	Human	Bevacizumab	3.099 × 10^4^	4.457 × 10^−^^5^	1.438 × 10^−^^9^

ECD: extracellular domain; *k_a_*: association rate constant; *k_d_*: dissociation rate constant; *K*_D_: equilibrium dissociation rate constant.

**Table 2:** Pharmacokinetic (PK) parameters estimated by noncompartmental analysis after a single intravenous (i.v.) injection of 10 mg/kg IDB0076 or bevacizumab into Sprague–Dawley (SD) rats according to the assay methods.

Method	Drug	AUC_all_ (μg/mL*day)	AUC_inf_ (μg/mL*day)	t_1/2_ (days)	C_0_ (μg/mL)	CL (mL/day/kg)	V_ss_ (mL/kg)
VEGFA binding	Bevacizumab	886.5 ± 71.8	1769.5 ± 110.4	14.7 ± 1.8	235.8 ± 4.4	5.7 ± 0.3	122.1 ± 13.0
	IDB0076	868.1 ± 61.6	1722.5 ± 173.1	14.7 ± 2.0	264.1 ± 4.6	6.1 ± 0.7	122.2 ± 11.8
Dual-targeting	IDB0076	947.9 ± 104.4	1384.2 ± 100.7	9.5 ± 0.6	270.8 ± 5.7	7.4 ± 0.5	99.4 ± 13.9

Values are expressed as mean ± standard deviation (*n* = 5). AUC_all_: area under the time–serum concentration curve from time zero to the last quantifiable observation, AUC_inf_: area under the time–serum concentration curve from time zero to infinity, t_1/2_: elimination half-life, C_0_: initial serum concentration at time zero, CL: clearance, and V_ss_: the volume of distribution in the steady state.

**Table 3:** Blood chemistry in cynomolgus monkeys that received IDB0076.

**Sex**	**Dose**	**AST (IU/L)**			**ALT (IU/L)**			**ALP (IU/L)**			**GGT (IU/L)**		
		**Pre**	**End of** **dosing**	**End of recovery**	**Pre**	**End of dosing**	**End of recovery**	**Pre**	**End of dosing**	**End of recovery**	**Pre**	**End of dosing**	**End of recovery**
Males	2 mg/kg	66	53	N/A	54	43	N/A	1912	2038	N/A	70	80	N/A
	10 mg/kg	30	55	N/A	34	47	N/A	1223	1102	N/A	58	59	N/A
	50 mg/kg	27	44	N/A	21	21	N/A	1166	1265	N/A	67	97	N/A
	50 mg/kg-recovery	34	34	27	33	24	21	1178	1107	1412	48	57	52
Females	2 mg/kg	43	50	N/A	83	76	N/A	694	613	N/A	56	54	N/A
	10 mg/kg	44	35	N/A	108	44	N/A	554	466	N/A	52	52	N/A
	50 mg/kg	30	46	N/A	40	45	N/A	677	882	N/A	45	54	N/A
	50 mg/kg-recovery	42	104	34	71	53	35	822	1124	674	43	53	38
**Sex**	**Dose**	**Creatinine (mg/dL)**			**CK (IU/L)**			**Total bilirubin (mg/dL)**			**Urea nitrogen (mg/dL)**		
		**Pre**	**End of dosing**	**End of recovery**	**Pre**	**End of dosing**	**End of recovery**	**Pre**	**End of dosing**	**End of recovery**	**Pre**	**End of dosing**	**End of recovery**
Males	2 mg/kg	0.66	0.60	N/A	377	198	N/A	0.07	0.10	N/A	34.3	30.3	N/A
	10 mg/kg	0.69	0.66	N/A	254	452	N/A	0.17	0.25	N/A	19.4	18.4	N/A
	50 mg/kg	0.85	0.82	N/A	188	156	N/A	0.11	0.08	N/A	22.0	22.6	N/A
	50 mg/kg-recovery	0.58	0.58	0.58	174	111	118	0.12	0.13	0.08	17.8	18.2	21.1
Females	2 mg/kg	0.53	0.49	N/A	199	254	N/A	0.21	0.12	N/A	16.3	22.0	N/A
	10 mg/kg	0.65	0.59	N/A	177	160	N/A	0.14	0.13	N/A	18.2	17.4	N/A
	50 mg/kg	0.71	0.78	N/A	378	133	N/A	0.17	0.18	N/A	23.7	22.5	N/A
	50 mg/kg-recovery	0.60	0.63	0.64	187	529	364	0.15	0.12	0.13	16.8	16.7	15.4
**Sex**	**Dose**	**Glucose (mg/dL)**			**IP (mg/dL)**			**TP (g/dL)**			**Albumin (g/dL)**		
		**Pre**	**End of dosing**	**End of recovery**	**Pre**	**End of dosing**	**End of recovery**	**Pre**	**End of dosing**	**End of recovery**	**Pre**	**End of dosing**	**End of recovery**
Males	2 mg/kg	78	83	N/A	4.98	4.80	N/A	7.6	7.0	N/A	3.8	3.5	N/A
	10 mg/kg	75	72	N/A	6.50	4.81	N/A	7.6	7.2	N/A	4.1	3.7	N/A
	50 mg/kg	85	78	N/A	5.67	3.12	N/A	6.8	7.8	N/A	3.6	3.5	N/A
	50 mg/kg-recovery	85	83	79	5.53	3.79	4.36	7.9	8.2	7.5	4.1	3.8	3.7
Females	2 mg/kg	78	73	N/A	4.99	3.46	N/A	7.4	7.0	N/A	4.4	4.0	N/A
	10 mg/kg	59	63	N/A	6.00	4.19	N/A	7.6	7.1	N/A	3.6	3.5	N/A
	50 mg/kg	66	61	N/A	5.36	3.46	N/A	7.6	7.6	N/A	4.0	3.5	N/A
	50 mg/kg-recovery	80	76	71	4.65	2.61	4.33	7.8	8.0	7.8	4.3	3.5	4.1
Sex	**Dose**	**Globulin (g/dL)**			**Albumin/globulin ratio**			**TG (mg/dL)**			**Total cholesterol (mg/dL)**		
		**Pre**	**End of dosing**	**End of recovery**	**Pre**	**End of dosing**	**End of recovery**	**Pre**	**End of Dosing**	**End of recovery**	**Pre**	**End of dosing**	**End of Recovery**
Males	2 mg/kg	3.8	3.5	N/A	1.00	1.00	N/A	103	67	N/A	123	120	N/A
	10 mg/kg	3.5	3.5	N/A	1.17	1.06	N/A	29	24	N/A	158	172	N/A
	50 mg/kg	3.2	4.3	N/A	1.13	0.81	N/A	37	46	N/A	168	177	N/A
	50 mg/kg-recovery	3.8	4.4	3.8	1.08	0.86	0.97	32	18	38	135	147	128
Females	2 mg/kg	3.0	3.0	N/A	1.47	1.33	N/A	39	96	N/A	92	87	N/A
	10 mg/kg	4.0	3.6	N/A	0.90	0.97	N/A	63	35	N/A	156	162	N/A
	50 mg/kg	3.6	4.1	N/A	1.11	0.85	N/A	40	57	N/A	127	223	N/A
	50 mg/kg-recovery	3.5	4.5	3.7	1.23	0.78	1.11	47	31	26	148	196	148

ALP: alkaline phosphatase, ALT: alanine transaminase, AST: aspartate transaminase, CK: creatine kinase, GGT: γ-glutamyltransferase, IP: inorganic phosphorus, N/A: not applicable, TG: triglycerides, TP: total protein.

**Table 4:** A summary of histopathological findings in cynomolgus monkeys that received IDB0076.

Sex	Dose	Organ/Tissue	Findings	Severity
**Light-microscopic findings**				
Males	2 mg/kg	N/A	-	-
	10 mg/kg	Femur	Decreased number of primary trabecula bone Increased thickness of physis	Slight Slight
	50 mg/kg	Femur	Decreased number of primary trabecula bone Increased thickness of physis	Slight Slight
		Kidney	Enlargement of glomeruli	Very slight
	50 mg/kg-recovery	Femur	-	-
Females	2 mg/kg	N/A	-	-
	10 mg/kg	N/A	-	-
	50 mg/kg	Kidney	Enlargement of glomeruli	Very slight
	50 mg/kg-recovery	N/A	-	-
**Electron-microscopic findings**				
Males	2 mg/kg	Kidney (Glomerulus)	-	-
	50 mg/kg		Hypertrophy of podocytes Shortening of foot process in podocytes Retention of small/large vesicles in capillaries	Slight Very slight Slight
	50 mg/kg-recovery		Retention of vesicles in capillaries	Very slight
Females	2 mg/kg		-	-
	50 mg/kg		Hypertrophy of podocytes Shortening of foot process in podocytes Retention of small/large vesicles in capillaries Granular/vesicular material in mesangial cells	Slight Very slight Slight Very slight
	50 mg/kg-recovery		Retention of vesicles in capillaries	Very slight

“-” means no abnormal change.

**Table 5:** Urinalysis in cynomolgus monkeys that received IDB0076.

**Sex**	**Dose**	**Color**			**pH**			**Ketones (mg/dL)**			**Bilirubin (mg/dL)**			**Occult blood (mg/dL)**		
		**Pre**	**End of dosing**	**End of recovery**	**Pre**	**End of dosing**	**End of recovery**	**Pre**	**End of dosing**	**End of recovery**	**Pre**	**End of dosing**	**End of recovery**	**Pre**	**End of dosing**	**End of recovery**
Males	2 mg/kg	0	0	N/A	8.5	8.5	N/A	0	0	N/A	0	0	N/A	0	0	N/A
	10 mg/kg	0	0	N/A	8.5	8.5	N/A	0	0	N/A	0	0	N/A	0	0	N/A
	50 mg/kg	0	0	N/A	8.5	8.5	N/A	0	0	N/A	0	0	N/A	0	0	N/A
	50 mg/kg-recovery	0	0	0	8.5	8.5	8.5	0	0	0	0	0	0	0	0	0
Females	2 mg/kg	0	0	N/A	8.5	8.5	N/A	0	0	N/A	0	0	N/A	0	0	N/A
	10 mg/kg	0	0	N/A	8.5	8.5	N/A	0	0	N/A	0	0	N/A	0	0	N/A
	50 mg/kg	0	0	N/A	8.5	8.5	N/A	0	0	N/A	0	0	N/A	0	0.015	N/A
	50 mg/kg-recovery	0	0	0	8.5	8.5	8.5	0	0	0	0	0	0	0	0	0
**Sex**	**Dose**	**Urobilinogen (Ehrlich units/dL)**			**Urine volume (mL)**			**Specific gravity**			**Protein (mg/dL)**			**Glucose (mg/dL)**		
		**Pre**	**End of dosing**	**End of recovery**	**Pre**	**End of dosing**	**End of recovery**	**Pre**	**End of dosing**	**End of recovery**	**Pre**	**End of dosing**	**End of recovery**	**Pre**	**End of dosing**	**End of recovery**
Males	2 mg/kg	0	0	N/A	78	75	N/A	1.024	1.025	N/A	0.2	2.4	N/A	0	2	N/A
	10 mg/kg	0	0	N/A	52	88	N/A	1.021	1.016	N/A	6.7	8.0	N/A	4	5	N/A
	50 mg/kg	0	0	N/A	80	122	N/A	1.021	1.017	N/A	3.8	13.0	N/A	3	8	N/A
	50 mg/kg-recovery	0	0	0	96	106	116	1.016	1.015	1.018	1.6	4.2	0.5	4	6	1
Females	2 mg/kg	0	0	N/A	126	190	N/A	1.014	1.013	N/A	3.1	3.6	N/A	2	6	N/A
	10 mg/kg	0	0	N/A	108	164	N/A	1.012	1.009	N/A	4.9	14.4	N/A	1	5	N/A
	50 mg/kg	0	0	N/A	108	78	N/A	1.012	1.015	N/A	1.7	2.7	N/A	3	4	N/A
	50 mg/kg-recovery	0	0	0	88	182	96	1.018	1.010	1.014	8.7	36.8	14.0	5	8	6
**Sex**	**Dose**	**Na (mEq/L)**			**K (mEq/L)**			**Cl (mEq/L)**								
		**Pre**	**End of dosing**	**End of recovery**	**Pre**	**End of dosing**	**End of recovery**	**Pre**	**End of dosing**	**End of recovery**						
Males	2 mg/kg	15	20	N/A	100.7	112.0	N/A	78	78	N/A						
	10 mg/kg	23	27	N/A	75.7	39.3	N/A	66	56	N/A						
	50 mg/kg	23	21	N/A	75.0	47.1	N/A	63	40	N/A						
	50 mg/kg-recovery	16	11	13	53.5	52.3	74.4	41	29	47						
Females	2 mg/kg	20	15	N/A	21.0	39.7	N/A	47	33	N/A						
	10 mg/kg	42	19	N/A	30.0	22.8	N/A	46	22	N/A						
	50 mg/kg	24	30	N/A	38.8	45.0	N/A	31	20	N/A						
	50 mg/kg-recovery	36	25	26	37.0	21.6	27.0	42	31	27						

Color. 0: normal, 1: abnormal. Cl: chloride; Na: sodium; K: potassium; N/A: not applicable.

## Supplementary Materials

The following are available online at https://www.mdpi.com/2218-273X/10/6/919/s1, Figure S1: IDB0076 downregulates the cell surface levels of NRP1 in PANC-1 cells, Table S1: Hematology in cynomolgus monkeys that received IDB0076, Table S2: Absolute and relative weight in cynomolgus monkeys that received IDB0076.
