# Supplementary material for: Preclinical Efficacy and Safety of an Anti-Human VEGFA and Anti-Human NRP1 Dual-Targeting Bispecific Antibody (IDB0076)
Source: Biomolecules. 2020 Jun 17;10(6):919. doi: 10.3390/biom10060919 (PMC7356919; doi:10.3390/biom10060919)
Supplement: Supplementary file 1 [file biomolecules-10-00919-s001.pdf]

biomolecules

Supplementary Information

Preclinical Efficacy and Safety of an Anti-human VEGFA and Anti-human NRP1 Dual-targeting Bispecific Antibody (IDB0076)

Jong-Hee Ko, Hyuk-Sang Kwon, Bomin Kim, Gihong Min, Chorong Shin, Seok-Woo Yang, Seong Wook Lee, Youngmin Lee, Dahae Hong, and Yong-Sung Kim

Supplementary Materials and Methods

A NRP1 Internalization Assay

PANC-1 cells (cat. # CRL-1469, ATCC) grown overnight on 8-well chamber slide (Ibidi) were washed, incubated in serum-free DMEM media for 30 minutes at 37 °C, and then treated with 2 μM IDB0076 or bevacizumab for 1 h at 4 °C or 37 °C. The slides were stained with an anti-neuropilin 1 antibody (cat. # ab81321, Abcam) and an anti-human IgG1 PE-conjugated antibody (cat. # IC002P, R&D systems). The secondary antibody was a goat anti-rabbit IgG Alexa Fluor 594-conjugated antibody (cat. # A11037, Thermo Fisher). Images were captured via confocal microscopy (Carl Zeiss) and were subjected to Zen 2.3 Blue edition analysis (Carl Zeiss).

Supplementary Figure S1

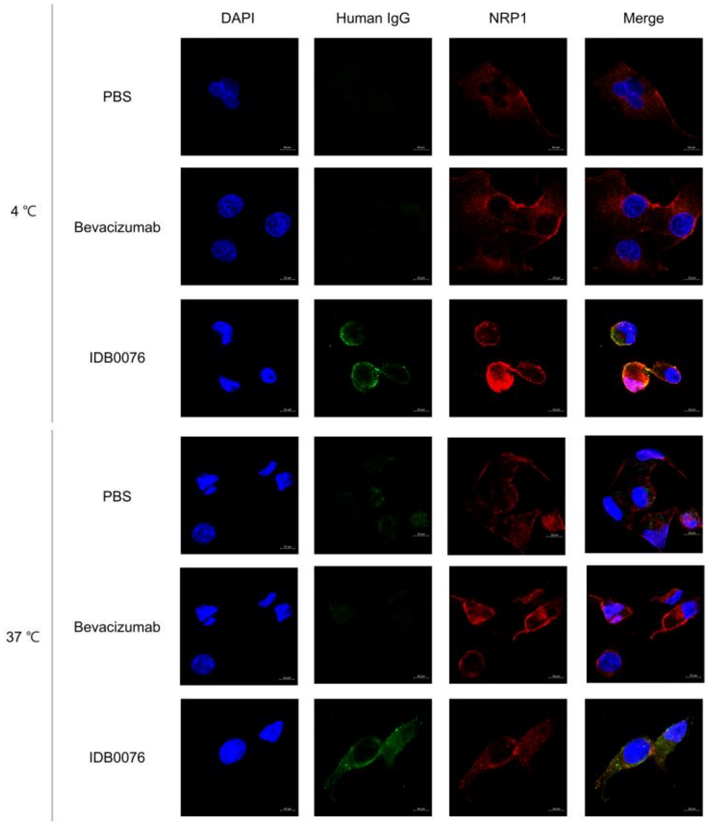

IDB0076 downregulates the cell surface levels of NRP1 in PANC-1 cells. Colocalization with cell-surface expressed NRP1 (red) at 4 °C (upper panels) and NRP1-mediated internalization at 37 °C (bottom panels) of IDB0076 in PANC-1 cells, assessed by confocal fluorescence microscopy. The cells were treated with IDB0076 or bevacizumab (2 μM) for 1 h at 4 °C or 37 °C. Nuclei were co-stained with DAPI (blue). Magnification: ×80, scale bar: 10 μm.

Supplementary Table S1

Hematology in cynomolgus monkeys that received IDB0076

Sex	Dose	RBC (10^6/μL)			HGB (g/dL)			HCT (%)			MCV (fL)		
		Pre	End of dosing	End of recovery	Pre	End of dosing	End of recovery	Pre	End of dosing	End of recovery	Pre	End of dosing	End of recovery
Males	2 mg/kg	5.59	5.86	N/A	13.8	14.3	N/A	43.8	45.1	N/A	78.4	77.0	N/A
	10 mg/kg	6.14	6.19	N/A	13.7	13.9	N/A	42.7	43.5	N/A	69.5	70.3	N/A
	50 mg/kg	5.53	6.13	N/A	13.1	14.2	N/A	40.3	44.5	N/A	72.9	72.6	N/A
	50 mg/kg-recovery	6.14	6.62	6.27	12.8	13.7	13.2	41.8	43.9	42.5	68.1	66.3	67.8
Females	2 mg/kg	5.38	5.39	N/A	12.9	13.2	N/A	40.2	41.2	N/A	74.7	76.4	N/A
	10 mg/kg	5.77	6.27	N/A	13.4	14.4	N/A	42.1	44.6	N/A	73.0	71.1	N/A
	50 mg/kg	5.09	5.94	N/A	12.5	14.5	N/A	38.7	44.1	N/A	76.0	74.2	N/A
	50 mg/kg-recovery	5.19	5.94	5.70	12.7	14.3	13.8	40.3	45.1	42.7	77.6	75.9	74.9

Sex	Dose	MCH (pg)			MCHC (g/dL)			PLT (10^3/μL)			RET (%)		
		Pre	End of dosing	End of recovery	Pre	End of dosing	End of recovery	Pre	End of dosing	End of recovery	Pre	End of dosing	End of recovery
Males	2 mg/kg	24.7	24.4	N/A	31.5	31.7	N/A	342	323	N/A	0.73	0.62	N/A
	10 mg/kg	22.3	22.5	N/A	32.1	32.0	N/A	285	298	N/A	0.44	0.71	N/A
	50 mg/kg	23.7	23.2	N/A	32.5	31.9	N/A	332	277	N/A	0.42	0.79	N/A
	50 mg/kg-recovery	20.8	20.7	21.1	30.6	31.2	31.1	314	298	284	0.75	0.57	0.80
Females	2 mg/kg	24.0	24.5	N/A	32.1	32.0	N/A	505	446	N/A	0.37	0.38	N/A
	10 mg/kg	23.2	23.0	N/A	31.8	32.3	N/A	321	326	N/A	0.55	0.44	N/A
	50 mg/kg	24.6	24.4	N/A	32.3	32.9	N/A	389	227	N/A	0.52	0.69	N/A
	50 mg/kg-recovery	24.5	24.1	24.2	31.5	31.7	32.3	407	284	401	1.34	1.25	0.80

Sex	Dose	RET. (10^9/μL)			WBC (10^3/μL)			Neutro. (10^3/μL)			Lymph. (10^3/μL)		
		Pre	End of dosing	End of recovery	Pre	End of dosing	End of recovery	Pre	End of dosing	End of recovery	Pre	End of dosing	End of recovery
Males	2 mg/kg	40.8	36.3	N/A	6.97	6.61	N/A	2.38	1.70	N/A	3.92	4.03	N/A
	10 mg/kg	27.0	43.9	N/A	5.46	5.57	N/A	1.77	1.61	N/A	3.42	3.61	N/A
	50 mg/kg	23.2	48.4	N/A	9.64	11.02	N/A	4.88	3.62	N/A	4.11	6.41	N/A
	50 mg/kg-recovery	46.0	37.7	50.2	18.43	20.43	23.60	3.56	5.00	3.69	13.05	13.46	17.10
Females	2 mg/kg	19.9	20.5	N/A	9.08	12.81	N/A	5.90	8.23	N/A	2.62	3.84	N/A
	10 mg/kg	31.7	27.6	N/A	14.75	11.84	N/A	9.73	5.89	N/A	3.95	5.19	N/A
	50 mg/kg	26.5	41.0	N/A	8.20	5.76	N/A	4.33	1.15	N/A	3.51	4.09	N/A
	50 mg/kg-recovery	69.5	74.3	45.6	6.77	10.05	9.21	2.97	1.87	2.21	3.45	7.42	6.54

Sex	Dose	Mono. (10^3/μL)			Eosino. (10^3/μL)			Baso. (10^3/μL)			Neutro. (%)		
		Pre	End of dosing	End of recovery	Pre	End of dosing	End of recovery	Pre	End of dosing	End of recovery	Pre	End of dosing	End of recovery
Males	2 mg/kg	0.53	0.64	N/A	0.14	0.23	N/A	0.00	0.01	N/A	34.2	25.6	N/A
	10 mg/kg	0.25	0.25	N/A	0.02	0.09	N/A	0.00	0.01	N/A	32.4	28.9	N/A
	50 mg/kg	0.52	0.77	N/A	0.12	0.20	N/A	0.01	0.02	N/A	50.7	32.8	N/A
	50 mg/kg-recovery	1.69	1.67	2.63	0.11	0.28	0.15	0.02	0.02	0.03	19.3	24.4	15.7
Females	2 mg/kg	0.50	0.64	N/A	0.05	0.09	N/A	0.01	0.01	N/A	64.9	64.2	N/A
	10 mg/kg	1.00	0.66	N/A	0.06	0.09	N/A	0.01	0.01	N/A	65.9	49.7	N/A
	50 mg/kg	0.30	0.34	N/A	0.06	0.17	N/A	0.00	0.01	N/A	52.8	19.9	N/A
	50 mg/kg-recovery	0.34	0.58	0.43	0.01	0.17	0.03	0.00	0.01	0.00	43.9	18.6	24.0

Sex	Dose	Lymph. (%)			Mono. (%)			Eosino. (%)			Baso.(%)		
		Pre	End of dosing	End of recovery	Pre	End of dosing	End of recovery	Pre	End of dosing	End of recovery	Pre	End of dosing	End of recovery
Males	2 mg/kg	56.2	61.0	N/A	7.6	9.7	N/A	2.0	3.5	N/A	0.0	0.2	N/A

	10 mg/kg	62.3	64.8	N/A	4.6	4.5	N/A	0.4	1.6	N/A	0.0	0.2	N/A
	50 mg/kg	42.6	58.2	N/A	5.4	7.0	N/A	1.2	1.8	N/A	0.1	0.2	N/A
	50 mg/kg-recovery	70.8	65.9	72.5	9.2	8.2	11.1	0.6	1.4	0.6	0.1	0.1	0.1
Females	2 mg/kg	28.9	30.0	N/A	5.5	5.0	N/A	0.6	0.7	N/A	0.1	0.1	N/A
	10 mg/kg	26.8	43.8	N/A	6.8	5.6	N/A	0.4	0.8	N/A	0.1	0.1	N/A
	50 mg/kg	42.8	71.0	N/A	3.7	5.9	N/A	0.7	3.0	N/A	0.0	0.2	N/A
	50 mg/kg-recovery	51.0	73.8	71.0	5.0	5.8	4.7	0.1	1.7	0.3	0.0	0.1	0.0

Sex	Dose	PT (s)			APTT (s)		
		Pre	End of dosing	End of recovery	Pre	End of dosing	End of recovery
Males	2 mg/kg	10.3	10.6	N/A	16.5	17.2	N/A
	10 mg/kg	9.8	9.9	N/A	16.1	16.2	N/A
	50 mg/kg	9.8	9.7	N/A	16.2	17.2	N/A
	50 mg/kg-recovery	10.0	9.9	9.8	19.9	19.7	19.5
Females	2 mg/kg	9.9	9.8	N/A	21.3	20.0	N/A
	10 mg/kg	9.8	10.0	N/A	20.4	20.4	N/A
	50 mg/kg	10.2	9.9	N/A	22.1	22.7	N/A
	50 mg/kg-recovery	10.3	10.1	10.2	16.5	18.3	16.5

RBC: erythrocyte count, HGB: hemoglobin concentration, HCT: hematocrit value, MCV: mean corpuscular volume, MCH: mean corpuscular hemoglobin, MCHC: mean corpuscular hemoglobin concentration, PLT: platelet count, RET.: reticulocyte count, WBC: leukocyte count, Neutro.: neutrophil count, Lymph.: lymphocyte count, Mono.: monocyte count, Eosino.: eosinophil count, Baso.: basophil count, PT: prothrombin time, APTT: activated partial thromboplastin time

Supplementary Table S2

Absolute and relative organ weights in cynomolgus monkeys that received IDB0076

Absolute organ weights							
**Sex**	**Dose**	**Pituitary (mg)**	**Thymus (g)**	**Spleen (g)**	**Brain (g)**	**Heart (g)**	**Lung (g)**
Males	2 mg/kg	43	2.8	2.8	60.3	10.4	12.3
	10 mg/kg	29	1.5	3.3	67.3	9.8	11.0
	50 mg/kg	43	2.1	3.8	69.3	10.6	12.5
	50 mg/kg-recovery	40	3.7	5.4	66.6	8.3	13.7
Females	2 mg/kg	49	1.6	3.4	61.4	9.1	13.4
	10 mg/kg	56	2.1	3.3	57.9	9.9	10.7
	50 mg/kg	66	1.2	5.9	59.4	8.4	14.2
	50 mg/kg-recovery	59	1.6	2.1	66.5	9.4	14.4
**Sex**	**Dose**	**Liver (g)**	**Kidneys (g)**	**Testes (g)**	**Epididymides (g)**	**Ovaries (g)**	**Uterus (g)**
Males	2 mg/kg	56.6	10.7	1.8	0.6	N/A	N/A
	10 mg/kg	44.3	11.7	1.3	0.4	N/A	N/A
	50 mg/kg	69.9	12.2	1.7	0.7	N/A	N/A
	50 mg/kg-recovery	57.2	12.1	2.0	0.7	N/A	N/A
Females	2 mg/kg	68.0	12.7	N/A	N/A	0.15	4.0
	10 mg/kg	49.8	9.7	N/A	N/A	0.15	7.1
	50 mg/kg	49.4	10.2	N/A	N/A	0.25	5.4
	50 mg/kg-recovery	53.6	10.5	N/A	N/A	0.33	5.4

Relative organ weights							
**Sex**	**Dose**	**Pituitary (mg/kg)**	**Thymus (g/kg)**	**Spleen (g/kg)**	**Brain (g/kg)**	**Heart (g/kg)**	**Lung (g/kg)**
Males	2 mg/kg	14.9	0.97	0.97	20.87	3.60	4.26
	10 mg/kg	11.3	0.59	1.29	26.29	3.83	4.30

	50 mg/kg	14.0	0.68	1.24	22.57	3.45	4.07
	50 mg/kg-recovery	15.2	1.40	2.05	25.23	3.14	5.19
Females	2 mg/kg	17.4	0.57	1.21	21.85	3.24	4.77
	10 mg/kg	20.2	0.76	1.19	20.90	3.57	3.86
	50 mg/kg	24.9	0.45	2.23	22.42	3.17	5.36
	50 mg/kg-recovery	21.8	0.59	0.77	24.54	3.47	5.31
**Sex**	**Dose**	**Liver (g/kg)**	**Kidneys (g/kg)**	**Testes (g/kg)**	**Epididymides (g/kg)**	**Ovaries (g/kg)**	**Uterus (g/kg)**
Males	2 mg/kg	19.58	3.70	0.62	0.21	N/A	N/A
	10 mg/kg	17.30	4.57	0.51	0.16	N/A	N/A
	50 mg/kg	22.77	3.97	0.55	0.23	N/A	N/A
	50 mg/kg-recovery	21.67	4.58	0.76	0.27	N/A	N/A
Females	2 mg/kg	24.20	4.52	N/A	N/A	0.053	1.42
	10 mg/kg	17.98	3.50	N/A	N/A	0.054	2.56
	50 mg/kg	18.64	3.85	N/A	N/A	0.094	2.04
	50 mg/kg-recovery	19.78	3.87	N/A	N/A	0.122	1.99

N/A: not applicable
